# Rethinking animal attrition in preclinical research: Expressing causal mechanisms of selection bias using directed acyclic graphs

**DOI:** 10.1177/0271678X241275760

**Published:** 2024-08-20

**Authors:** Anja Collazo, Hans-Georg Kuhn, Tobias Kurth, Marco Piccininni, Jessica L Rohmann

**Affiliations:** 1 BIH QUEST Center for Responsible Research, Berlin Institute of Health, Berlin, Germany; 2 Institute of Public Health, Charité – Universitätsmedizin Berlin, Berlin, Germany; 3 Institute for Neuroscience and Physiology, Department of Clinical Neuroscience, 3570 University of Gothenburg, Gothenburg, Sweden; 4 Center for Stroke Research Berlin, Charité – Universitätsmedizin Berlin, Berlin, Germany; 5 Scientific Directorate, Max Delbrück Center for Molecular Medicine in the Helmholtz Association, Berlin, Germany

**Keywords:** Animal attrition, collider stratification bias, internal validity, preclinical research, selection bias

## Abstract

Animal attrition in preclinical experiments can introduce bias in the estimation of causal treatment effects, as the treatment-outcome association in surviving animals may not represent the causal effect of interest. This can compromise the internal validity of the study despite randomization at the outset. Directed Acyclic Graphs (DAGs) are useful tools to transparently visualize assumptions about the causal structure underlying observed data. By illustrating relationships between relevant variables, DAGs enable the detection of even less intuitive biases, and can thereby inform strategies for their mitigation. In this study, we present an illustrative causal model for preclinical stroke research, in which animal attrition induces a specific type of selection bias (i.e., collider stratification bias) due to the interplay of animal welfare, initial disease severity and negative side effects of treatment. Even when the treatment had no causal effect, our simulations revealed substantial bias across different scenarios. We show how researchers can detect and potentially mitigate this bias in the analysis phase, even when only data from surviving animals are available, if knowledge of the underlying causal process that gave rise to the data is available. Collider stratification bias should be a concern in preclinical animal studies with severe side effects and high post-randomization attrition.

## Introduction

The central goal of preclinical research is to determine whether a given treatment has a causal effect on a specific physiological outcome in animals and to quantify its impact. Ideally, researchers would like to observe the outcome in the same animal at the same moment under identical laboratory and environmental conditions, only altering the individual animal’s treatment status. The unobserved outcome that the animal “would have experienced” is known as the “counterfactual outcome”.^1^ However, without the ability to travel through time, making such comparisons remains a thought experiment. Since it is not possible to observe the two counterfactual outcomes of a single animal in the real world, researchers instead target an average causal effect for a group of animals and rely on the comparison between treatment and control groups of animals, which is directly observable.

Yet, to derive internally valid estimates, it is important to ensure that, aside from treatment, everything else at the group level is as similar as possible between the two groups of animals. If the groups systematically differ in terms of the (counterfactual) risk for the outcome due to different baseline characteristics, the comparison becomes invalid. In other words, the groups are not “exchangeable,” and the results may be biased.^1,2^

Preclinical researchers make use of randomized experiments to ensure, on average, this fair comparison between treatment groups.^3,4^ When researchers randomize animals into treatment groups and carry out randomization correctly, they expect the counterfactual risk for the outcome to be on average the same between the two groups of animals. This fulfills the condition of *exchangeability* between interventional groups. Without proper randomization of the treatment allocation, any observed difference between the two groups of animals may not necessarily represent the causal effect of interest. Instead, this may represent a mixing of the causal effect, if one is present, and other (non-causal) associations due to common causes that may predispose certain animals to both receiving the treatment and experiencing the outcome.^1^ This mixing, known as confounding, is well described in observational epidemiology^5^ and is also recognized in preclinical literature,^6–8^ although the terminology is not consistently used.

Another less-acknowledged threat to internal validity is selection bias. This term refers to a systematic error in the effect estimation that arises from the exclusion or inclusion of units into the sample.^1^ In the context of preclinical research, selection bias can arise, for example, when some animals initially randomized into an interventional group are ultimately excluded from the analysis because of sudden death or euthanasia to limit suffering due to deteriorating health during the course of the study. We refer to such losses as animal attrition.

Especially in animal models involving invasive procedures that produce substantial pain, distress, and/or impairment, animal attrition during the course of the experiment is expected, although not always transparently reported.^9–11^ A review of attrition frequencies in preclinical focal cerebral ischemia and cancer drug studies observed uneven sample sizes in 42% of all included experiments and that animal loss often exceeded 25% of all animals used when fully disclosed.^12^ Reported sample sizes in the treatment group tended to be smaller than in the control group, indicating higher attrition frequencies in the intervention groups.^12^

Implicitly, researchers often analyze preclinical data under the assumption that animal attrition occurs at random, as if the death of an animal was only related to a random event, such as a laboratory accident. However, if the attrition is not at random, and the experimental analysis is restricted to the subsample of surviving animals only, selection bias may arise.

In this work, we focus on a specific type of selection bias called collider stratification bias. This type of selection bias, unlike other types, is unique in that it can induce bias in the analysis even when the intervention has no effect on any individual (or other unit).^13,14^ Put simply, in the presence of collider stratification bias, an association can be observed *even when there is actually no true effect.* While collider stratification bias has been described in detail in other research fields, it remains absent from the discussion of preclinical research results, even though it likely poses a real threat to validity.

In this paper, we show how directed acyclic graphs (DAGs), a causal inference tool, can be used to transparently illustrate causal assumptions relevant to a given experimental set up and to detect potential scenarios in which collider stratification bias can arise. We detail a hypothetical example from the preclinical stroke research setting and present results from a simulation study, in which we quantify the magnitude of this bias and its impact on the obtained effect estimates under a variety of plausible parameter constellations.

## Methods

DAGs are visual representations of *a priori* hypothesized causal structures. They are widely used in observational epidemiological research to transparently depict the assumed causal relationships between relevant variables under study.^1,5,15–17^ Briefly, DAGs consist of nodes representing measured and unmeasured variables connected by arrows. An arrow extending from a “parent” to a “child” variable expresses a direct cause-effect relationship from the former to the latter in at least some units. Equally important is that the absence of an arrow between two nodes reflects the strong assumption of no cause-effect relationship between them for any unit.^1,5^ No information about the strength nor form of a relationship can be directly inferred from the arrows in a DAG. Since DAGs are acyclic, it is assumed that no variable can cause itself directly or via another variable; this would violate the temporality principle of cause preceding effect.

In a complete DAG, all direct effects among the variables as well as all common causes of any pair of variables included should be depicted.^1^ Increasingly implemented across research domains outside of epidemiology, DAGs have proven useful in diagnosing the presence of potential biases and guiding study design and analysis strategies in applied clinical research;^18–22^ however, only a few examples of the use of DAGs are found in preclinical research.^23,24^

In practice, researchers use DAGs to determine the sufficient adjustment set of variables needed to mitigate confounding or other biases to ultimately identify a causal contrast of interest. This is often achieved by identifying variables on the open non-causal path(s) between the exposure and the outcome in a DAG.^1,16^ If non-causal paths can be closed by design (e.g. through restriction) or analysis strategies (e.g. regression-based adjustment), then the causal effect of interest is identifiable.^1,16^

The usefulness of DAGs stems from inherent simplicity; these intuitive graphs can help researchers reason about design and analysis choices. Beneath the surface, these simple-looking diagrams encode conditional independencies between variables and connect simple graphical rules to complex identification theorems.^1,16^ DAGs have provided much-needed clarity to other fields looking to explain long-standing “paradoxes” that were difficult to reason about without an explicit causal framework.^16,25^ For further technical details, we refer readers to the extensive body of causal inference literature;^1,2,5,13,16,17^ our focus remains on bringing this tool to the preclinical context to help applied researchers better understand, detect, and mitigate a pervasive type of bias.

### Hypothetical preclinical example

**Figure 1.**
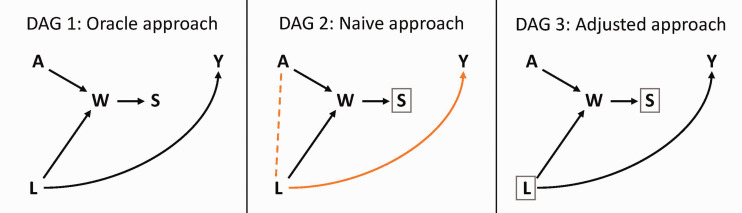
Exemplary directed acyclic graphs illustrating collider stratification bias in preclinical stroke research. Variables: *A*: exposure to treatment or control; *W*: animal welfare; *S*: survival status; *L*: initial infarct volume; *Y*: final infarct volume. In this hypothetical example, researchers are interested in estimating the causal effect of treatment *A* on final infarct volume *Y*. In DAG 1, hypothetically, we imagine that outcome information is available irrespective of the value of survival status (oracle approach). The collider path *A* → *W* ← *L* → *Y* is closed (black) and the average causal effect can be estimated correctly by comparing the outcome in the two groups. In DAG 2, measuring the outcome in surviving animals only (conditioning on *S* = 1, indicated by the box around *S*) induces a spurious association between *A* and *L*, opening a non-causal path between the exposure and the outcome (orange), and will result in biased effect estimation (naive approach). In DAG 3, adjusting for initial infarct volume (indicated by the box around *L*) closes the previously open non-causal path (now in black) and allows for the identification of the causal effect of interest (adjusted approach).

We use a DAG to depict how collider stratification bias can arise in the context of preclinical *in vivo* ischemic stroke research through differential animal attrition (Figure 1).

In interventional stroke experiments using an animal model, researchers often aim to derive the causal effect of a specific treatment on absolute (final) infarct size at the time point of outcome assessment. We use *A* to denote the assigned experimental group, with *A = *1 denoting the intervention group, *A = *0 denoting the control group, and *Y* to denote the final infarct volume in mm^3^. The causal effect of interest is therefore the average effect of *A* on *Y*, if all animals survive until the end of the study.

As shown in Figure 1, *Y* is also affected by the initial infarct volume, *L,*^26,27^ expressed as *L* → *Y*. We define initial infarct size as an early measure of the infarct severity determined by *in vivo* imaging after reperfusion and prior to any therapeutic intervention. The initial infarct size can vary substantially between animals depending on biological characteristics, the surgical methods used or the experimenter’s skill.^28–30^ By causing neurological deficits as well as inflammatory and immunological responses that alter physiological function, the initial infarct size, *L,* is also a contributing cause of the multidimensional composite variable animal welfare *W*,^31,32^ which is denoted in Figure 1 as the arrow *L* → *W.* As any treatment, *A,* can also have positive or negative side effects on animal welfare, we make this relationship explicit in the causal diagram with the arrow *A* → *W* (Figure 1). *W* is termed a collider variable on the path *A* → *W* ← *L* → *Y*, since the heads of the arrows connecting *A* → *W* and *L* → *W* “collide” into *W.*^1,13,14^ *S* represents the survival status with two possible states: *S = *1 indicates that the animal remained in the study until the outcome was assessed, *S = *0 indicates an unexpected loss or planned euthanasia of the animal during the course of the experiment, preventing the outcome assessment. The arrow *W* → *S* indicates that the animals’ survival status, *S,* depends on their welfare status; that is, *S* is a child of *W*. Indeed, animals maintaining an acceptable welfare level throughout the experiment remain in the final analytic sample, whereas animals that drop below a certain level of welfare do not survive because their conditions substantially deteriorate and lead to death or euthanasia for ethical reasons.

### Statistical analysis

For the *in silico* analyses, we translated the DAG in Figure 1 into a set of linear structural equations^16,17^ under the assumption of no effect of the treatment on the outcome for any animal. We considered different possible scenarios, generating the groups to have equal sizes of 5, 10 or 25 (corresponding to sample sizes, n_total_, of 10, 20 or 50).

We assumed treatment *A* to be assigned arbitrarily, and the initial infarct size, *L,* to be a normally distributed continuous variable representing an absolute volumetric measure of an ischemic infarct. We set the mean and standard deviation of *L* to 25 mm³ ± 5 mm³, reflecting an exemplary early infarct size in a middle cerebral artery occlusion (MCAO) model in rats that would be prominent enough to be detected through non-invasive imaging shortly after reperfusion.^27,33^

Values for welfare score, *W,* and the final infarct volume, *Y,* were simulated relying on the following linear structural equations:^17^

(1) $$Y=\beta_{0}+\beta_{1}\cdot A+\beta_{2}\cdot L+\varepsilon_{Y}$$

(2) $$W=\gamma_{0}+\gamma_{1}\cdot A+\gamma_{2}\cdot L+\varepsilon_{W}$$

The parameter values for coefficients $\beta_{1}$ (corresponding to *A → Y*), $\beta_{2}$ (*L → Y*), $\gamma_{1}$ (*A → W*), and $\gamma_{2}$ (*L → W*) express the causal effects of the respective parents on the child variables in the DAG. In our simulation, we set $\beta_{1}$ to zero in order to investigate the magnitude of collider stratification bias under the null (i.e., no effect of the intervention for any animal).^1^ We chose values for the other parameters in (1) in order to obtain realistic final infarct volumes. In a systematic review, O’Collins *et al.* reported an average absolute infarct volume of approximately 200 mm³ for an animal stroke model using Sprague-Dawley rats.^26^ Accordingly, we set $\beta_{0}$ to 0, since a final infarct volume without an initial infarct volume is not conceivable. We set $\beta_{2}$ to 8, which leads to an average final infarct volume of 200 mm^3^ according to (1). The exogenous error terms $\varepsilon_{Y}$ and $\varepsilon_{W}$ were assumed to be independent and normally distributed with a mean of zero and standard deviations of 10 and 2, respectively.

Since there is no standardized way of quantifying welfare,^32^ we generated a variable *W* with an arbitrary scale to reflect a quantified measure of welfare, with lower values indicating worse animal welfare. We set $\gamma_{0}$ to 0 and the parameter $\gamma_{2}$ to −1, reflecting the negative effect of initial infarct volume on animal welfare. We considered different scenarios in which the effect of the treatment on the welfare varied, indicating different possible treatment side effect profiles. Though often insufficiently reported, preclinical stroke studies tend to report higher animal attrition frequency for treatment groups compared to control groups, which might reflect drug toxicity.^12^ Therefore, we set $\gamma_{1}$ to {−1, −3, −6}, reflecting potential minor, moderate, and major negative side effects of treatment.

We captured the survival of an animal throughout the experiment in our simulations using the binary variable *S* (“Survival Status”), which was deterministically obtained from *W*. To capture the range of reported attrition frequencies for *in vivo* stroke research,^12^ we simulated the exclusion of animals having a welfare score below the 10th- (low attrition scenario), 25th- (moderate attrition scenario) or 50th- (high attrition scenario) percentiles of all animals in the simulated dataset. In practice, attrition frequencies may depend on the severity of the animal models or conditions particularly conducive (or detrimental) to survival.

Since we considered a continuous outcome, the treatment effect was estimated as the absolute difference between the mean final infarct volumes of animals in the treatment versus control groups. An effect estimate with a negative sign thus indicates a beneficial treatment effect.

For each scenario, we estimated the treatment effect using three approaches (in accordance with the identification strategies shown in Figure 1):

1. The “oracle” approach: the effect estimate was calculated as the difference in the mean final infarct volume between treated and untreated animals using all data points, including animals with *S* = 0. Obviously, this “all-knowing” approach is not feasible in the real world, since the variable *Y* is not observed for animals with *S* = 0; these animals did not survive to the time point of outcome assessment.
2. The “naive” approach: The treatment effect was estimated as the difference in the mean final infarct volume between treated and untreated groups *only* among those animals that survived until the time point of outcome measurement (restricting to *S = *1), mirroring real-world conditions.
3. The “adjusted” approach: The treatment effect estimate was obtained from a linear regression with final infarct volume as the dependent variable and treatment status and initial infarct volume as independent variables. The regression was fit *only* among animals that survived until the time point of outcome assessment (restricting to *S = *1), mirroring real-world conditions. The regression coefficient for treatment thus represents the estimated effect of the treatment among surviving animals, adjusted for *L*.

In total, we created 27 distinct scenarios with all possible constellations of the parameters (small, medium, or large sample size; minor, moderate, or major side effects; low, moderate, or high attrition). For each scenario, the simulation was repeated 10,000 times. We calculated the mean, the 2.5th, and 97.5th percentiles of the effect estimates obtained using the three aforementioned approaches (oracle, naive, adjusted) across the 10,000 runs.

We used R version 4.2.1 and RStudio version 2022.12.0 for all analyses and visualizations. The corresponding R code, figures, and tables can be retrieved from our Git repository (https://github.com/jrohmann/rethinking_animal_attrition).

## Results

Given the DAG 1 in Figure 1, both treatment and initial infarct volume affect welfare, and they are independent of each other. However, the complete-case analysis (naive approach, Figure 1, DAG 2) involves estimating the exposure-outcome association only among the stratum of surviving animals (*S = *1). This selection conditions on a child, *S*, of the collider, *W* (indicated by the box around *S* in Figure 1, DAG 2). The restriction to analyzing those data from surviving animals only thus induces a spurious association via a collider path, as shown by the orange dashed line in Figure 1, DAG 2.^5,13^ Thus, the measured association between *A* and *Y* in the data does not reflect the causal effect of interest, but instead a mixing between the effect of interest (if one exists) and an additional non-causal association introduced by the open path *A → W ←L → Y*.

From the assumed data generation mechanism (structural equations, distributional assumptions, and chosen parameters), it follows that animals have a higher probability of dying or being euthanized during the course of the study if they were in the treatment group (*A = *1) due to negative side effects or if they had large initial infarct volumes (*L*). Since having received treatment and having large initial infarct volumes are both causes of low welfare, among the surviving animals (*S = *1), treated animals are less likely to have large initial infarct volumes. Given that final infarct volume (*Y*) increases with the initial one (*L)*, smaller initial infarct volumes in the surviving treated animals result in smaller final infarct volumes compared with surviving control animals.

**Figure 2.**
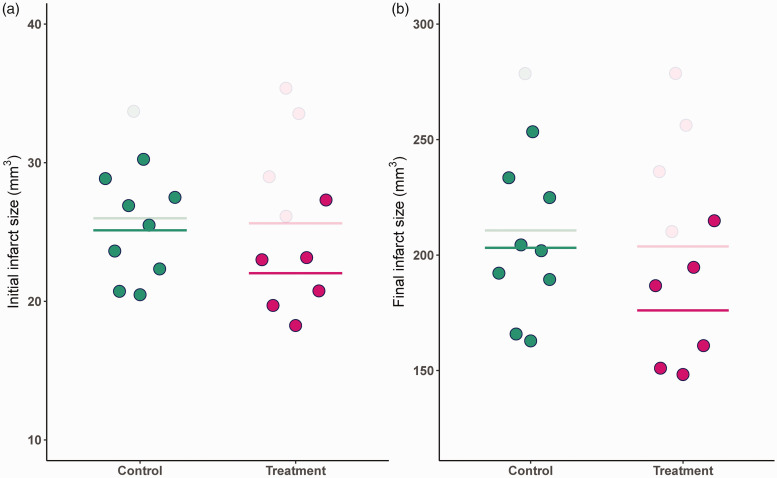
Hypothetical example illustrating the impact of collider stratification bias on mean initial and final infarct sizes using simulated data. Values for initial infarct volume, *L*, and final infarct volume, *Y*, are shown. The solid dots represent data from surviving animals (*S* = 1), and the solid lines represent the mean values for the underlying infarct size, *L*, and observed outcome, *Y*, in surviving animals of each by experimental group (naive approach). The semi-transparent dots represent values of censored animals (*S* = 0). The semi-transparent lines depict the means of *L* and *Y* in the hypothetical dataset without censoring (oracle approach). Panel (a): At study outset, both experimental groups show similar means for the initial infarct size *L* (semi-transparent line). Due to the underlying data generation mechanism, when considering only surviving animals, animals in the treatment group show, on average, smaller initial infarct sizes (solid points). Panel (b): Since *Y* decreases with decreasing *L*, the surviving animals of the treatment group show smaller final infarct sizes compared to surviving animals of the control group, despite absence of an actual causal effect.

Consequently, in such scenarios, the naive approach to effect estimation (i.e., complete case analysis of surviving animals) is biased in favor of the treatment because of the treatment’s negative side effects even in the absence of any causal treatment effect. This phenomenon is visualized in Figure 2. By comparing the initial infarct volume only among the *surviving* treated and untreated animals (as shown in Figure 2(a)), collider stratification bias can be detected. If this bias, which arises from the non-causal path opened by the selection on surviving animals only, is detected, measuring baseline variables on such a non-causal path and adjusting for them could represent a useful bias mitigation strategy.

**Figure 3.**
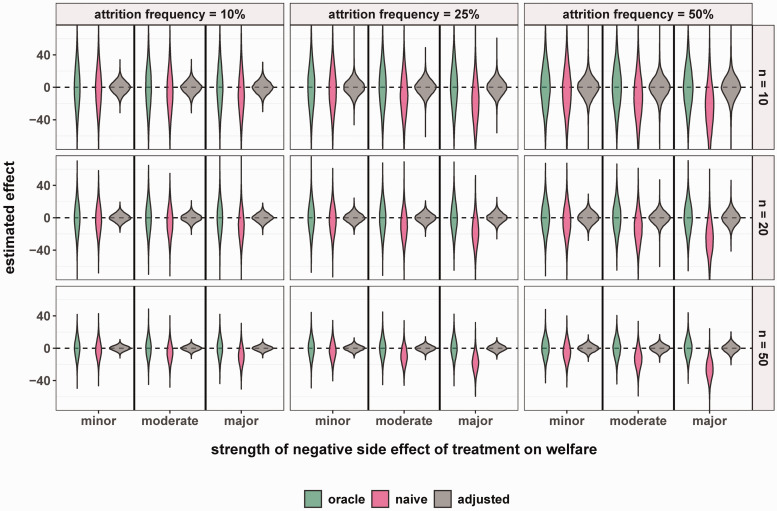
Simulation results: Three approaches for effect estimation under different attrition frequencies, strengths of side effects and sample sizes. Distribution of effect estimates for 10,000 simulated experiments for each of 27 scenarios created from the combination of different total sample sizes, n (10, 20 or 50), strength of side effects, $\gamma_{1}$ (major, moderate, minor) and attrition frequency (10%, 25%, 50%). Results are shown separately for the oracle approach (green): complete dataset E(Y | A = 1) - E(Y | A = 0); the naive approach (pink): with censoring of animals according to welfare, E(Y | A = 1, S = 1) - E(Y | A = 0, S = 1); and the adjusted approach (gray): with censoring of animals according to welfare and adjustment for initial infarct volume, E(Y | A = 1, S = 1, L = l) - E(Y | A = 0, S = 1, L = l). While the distributions of the estimates from the oracle approach (green) and the adjusted approach (gray) are centered approximately around the true causal effect of zero, the average effect estimate obtained by the naive approach (pink) appears to deviate substantially from zero in multiple scenarios.

The results from our simulation of 10,000 experiments for each parameter constellation in the assumed data generation mechanism are shown in Figure 3.

In 0.5% of the simulated experiments, the effect estimates for the naive and adjusted approach could not be calculated. For instance, in the scenario with very few animals (n_total_ = 10), high attrition (50%), and major side effects, these effect estimates could not be computed 10% of the time. This occurred because too few animals in one of the two experimental arms happened to survive in the experiment. We excluded these missing values from calculation of mean and quantiles of the effect estimates.

The mean causal effect estimate obtained using the oracle approach was approximately zero across all 27 scenarios (Figure 3 and Table 1). Indeed, as expected, this approach was unbiased for the targeted effect. In contrast, the mean effect estimates obtained using the naive approach differed from the true causal effect (equal to zero), indicating bias (Figure 3 and Table 1). This result shows that including only the surviving animals (*S* = 1, complete-case analysis) can induce an association between the treatment and the outcome merely due to the collider stratification bias. The magnitude of this bias varied widely across the different scenarios and increased with increasing attrition frequencies and side effect severity (lower $\gamma_{1}$ ). When the attrition rate was low (10%) and side effects were minor, the bias arising in the naive approach when n_total_ = 10 was −1.6 mm^3^ (2.5th and 97.5th percentiles: −49.6, 48.2) and −1.9 (−23.1, 19.9) when n_total_ = 50 (Table 1). With high attrition (50%) and major side effects, the quantified bias observed using this approach was as large as −24.5 mm^3^ (−83.2, 32.9) for n_total_ = 10 and −25.7 mm^3^ (−51.0, −1.2) for n_total_ = 50 (Table 1). While improving the precision of the effect estimates (less variability), increasing the sample size did not reduce the naive approach’s bias induced by collider stratification.

**Table 1.** Effect estimates obtained using three different approaches under different scenarios of sample size, attrition frequencies, and side effects.

Attrition frequencies (%)	Side effects, severity	Oracle approach: mean (mm³)	Oracle approach: 2.5% and 97.5% percentiles (mm³)	Naive approach: mean (mm³)	Naive approach: 2.5% and 97.5% quantiles (mm³)	Adjusted approach: mean (mm³)	Adjusted approach: 2.5% and 97.5% quantiles (mm³)
n = 10							
10	Minor	0.2	−50.7, 52.3	−1.6	−49.6, 48.2	−0.1	−14.3, 14.4
	Moderate	−0.5	−51.7, 51.0	−5.2	−53.8, 44.9	−0.1	−14.8, 14.5
	Major	0.2	−49.7, 51.1	−7.8	−56.6, 43.0	0.0	−14.4, 14.7
25	Minor	0.0	−51.3, 51.4	−2.9	−52.7, 46.2	0.0	−17.6, 17.7
	Moderate	0.2	−51.4, 50.5	−9.1	−59.9, 40.6	0.0	−17.9, 17.7
	Major	0.6	−51.0, 51.9	−17.4	−68.4, 34.3	0.1	−18.3, 19.0
50	Minor	0.2	−51.0, 51.7	−4.3	−59.7, 50.8	−0.1	−25.4, 25.0
	Moderate	0.3	−51.2, 52.0	−12.3	−70.3, 44.0	−0.3	−27.8, 25.2
	Major	0.0	−49.2, 51.4	−24.5	−83.2, 32.9	0.0	−29.4, 29.9
n = 20							
10	Minor	−0.1	−36.1, 36.7	−1.8	−35.7, 33.1	0.1	−9.5, 9.7
	Moderate	0.1	−36.5, 36.8	−5.0	−39.2, 29.8	−0.1	−9.8, 9.5
	Major	−0.2	−36.1, 36.5	−9.1	−43.7, 26.7	0.0	−9.7, 9.9
25	Minor	0.2	−35.6, 36.1	−2.9	−36.6, 30.5	0.0	−10.6, 10.8
	Moderate	−0.3	−36.1, 35.2	−9.2	−43.1, 25.6	−0.1	−10.9, 10.8
	Major	−0.1	−35.9, 35.9	−16.9	−52.5, 19.2	0.1	−11.2, 11.7
50	Minor	0.2	−36.0, 36.6	−4.3	−42.3, 32.9	0.0	−13.9, 13.8
	Moderate	0.1	−35.7, 36.1	−12.9	−51.4, 25.4	−0.1	−15.0, 14.5
	Major	0.0	−36.8, 35.7	−25.4	−67.4, 14.9	−0.1	−16.8, 16.8
n = 50							
10	Minor	0.1	−22.8, 23.5	−1.9	−23.1, 19.9	0.0	−5.9, 5.8
	Moderate	0.1	−22.9, 23.1	−5.3	−27.4, 16.2	0.0	−5.9, 5.9
	Major	0.0	−23.4, 23.5	−9.4	−32.0, 12.8	0.0	−5.9, 6.0
25	Minor	0.0	−22.9, 22.8	−3.3	−24.4, 18.2	0.0	−6.8, 6.6
	Moderate	0.0	−22.5, 22.8	−9.3	−30.5, 11.9	−0.1	−6.6, 6.6
	Major	0.0	−23.1, 23.1	−17.8	−40.4, 4.5	0.0	−7.0, 7.1
50	Minor	0.1	−22.8, 22.6	−4.4	−27.7, 18.4	0.0	−8.3, 7.9
	Moderate	0.0	−23.3, 22.8	−12.8	−36.4, 10.7	0.1	−8.5, 8.5
	Major	0.2	−22.6, 22.6	−25.7	−51.0, −1.2	0.1	−9.8, 9.7

Mean effect estimates, 2.5th, and 97.5th percentiles for 10,000 simulated experiments for 27 scenarios created from the combination of different total sample sizes, n (10, 20 or 50), strength of side effects, $\gamma_{1}$ (major, moderate, minor) and attrition frequencies (10%, 25%, 50%). Results are shown separately for the oracle approach: complete dataset, E(Y | A = 1) - E(Y | A = 0); the naive approach: with censoring of animals according to welfare, E(Y | A = 1, S = 1) - E(Y | A = 0, S = 1); and the adjusted approach: with censoring of animals according to welfare and adjustment for initial infarct volume, E(Y | A = 1, S = 1, L = l) - E(Y | A = 0, S = 1, L = l).

We note that while attrition frequencies were similar for both groups in the presence of minor side effects, this was not the case when moderate or large side effects were present. As expected, according to our data generation mechanism, severe side effects associated with the treatment led to higher attrition rates in the treatment group (Table 2).

**Table 2.** Attrition frequencies stratified by experimental group.

Side effects, severity	Total attrition frequencies (%)	Attrition frequency in treatment group (%)	Attrition frequency in control group (%)
Minor	10	12	8
	25	28	22
	50	54	46
Moderate	10	15	5
	25	34	16
	50	61	39
Major	10	18	2
	25	41	9
	50	71	29

Presentation of the attrition frequencies by experimental group under different parameter values for negative side effects $\gamma_{1}$ (A → W; minor, moderate, or major) and total attrition frequencies, (10%, 25% or 50%). Values of attrition frequency by group were obtained analytically.

When using the adjusted approach, we condition on *L*, blocking the non-causal path opened by the selection on *S* (*A → W ← L → Y*). In this way, the adjusted approach provides unbiased estimates for the causal effect conditional on *L* and *S* = 1. The conditional effect corresponds to the effect of the treatment among those surviving animals having the same initial infarct size (because this variable was conditioned upon).

In scenarios in which all relationships are linear, as described in (1) and (2), this conditional effect is equivalent to the marginal one (i.e., the average effect of the treatment among all animals), which was targeted in the oracle approach.

For this reason, the adjusted approach yielded mean effect estimates close to zero across all 27 scenarios (Table 1). Since the adjusted approach relies on the conditional distribution of the outcome, it not only provides an unbiased estimation of the true effect but is also more precise. For example, in the scenario with large sample size, high attrition, and major side effects, using the oracle approach, we obtained a mean effect estimate of 0.2 (−22.6, 22.6), while using the adjusted approach, a mean estimate of 0.1 (−9.8, 9.7).

## Discussion

In this work, we used DAGs to illustrate how differential animal attrition can induce collider stratification bias, a type of selection bias, in the estimation of causal effects in preclinical animal studies. We present a hypothetical example from preclinical stroke research in which the animal’s welfare is affected both by the treatment (i.e., negative side effects) and the initial infarct volume. Since the outcome, final infarct volume, is affected by the initial infarct size, we show how collider stratification bias can lead experimenters to erroneously conclude that a treatment has a beneficial effect even when no real effect exists. For simplicity, we assumed that the final infarct volume is a well-defined quantity even for animals lost to follow-up and considered the “oracle” approach to be the one of interest for the researcher. That is, the investigator’s interest lies in estimating the effect of the treatment on the final infarct volume in a world in which no animal dies during the study. This is a plausible causal effect of interest when all deaths are potentially preventable. For example, if all animals could survive until the moment of outcome assessment, but some are euthanized to prevent suffering. If this scenario is deemed unrealistic, other causal effects (e.g. conditional separable effects) should be targeted.^34^

The bias observed in our simulations arises from the fact that analyzing data only from the subset of animals that survived (“naive approach”) corresponds to implicitly conditioning on a (child of a) collider variable, thereby inducing a non-causal association between the treatment and the initial infarct size. We quantified the magnitude of the collider stratification bias in a simple model and showed that, being a systematic error, this bias cannot be reduced by increasing the number of animals in the experiment.

We illustrate that researchers can potentially mitigate this bias in the analysis phase, even when only data from surviving animals are available, by measuring and statistically adjusting for variable(s) on the open non-causal path (“adjusted approach”). This requires knowledge about the underlying causal structure that gave rise to the data, for which a DAG can be a useful visualization tool. In our simple hypothetical example, by measuring the initial infarct size (using e.g. magnetic resonance imaging) and including it as a covariate in the regression model fit using the data of the surviving animals, we showed how it is possible to mitigate the collider stratification bias even under high animal attrition and severe side effects.

Selection bias, though less intuitive than measurement error or confounding, has been described in the context of preclinical (stroke) research.^12,35–37^ We show how the use of a causal framework, specifically, through use of DAGs, can alert us to the presence of these biases and other threats to internal validity and inform the analysis in a transparent way. Collider stratification bias, unlike other selection biases, can lead to misleading effect estimates even when no effect is actually present.^1^ Therefore, it is plausible that erroneous decisions regarding the progression of treatments to confirmatory preclinical trials or first-in-human studies could be based on this bias, raising ethical concerns with regards to animal use and futile testing in patients.^38,39^ While we focus on the specific case in which the treatment has no effect in any animal, we emphasize that collider stratification can also induce bias by the same mechanism when the treatment has an effect on the outcome.^1^

### Limitations

While DAGs represent non-parametric models and the nature of collider stratification bias is agnostic to the specific type of functional relationship, we made several simplifying assumptions in our application.

First, we included a limited number of variables in our DAG to avoid overcomplication and focus on the simplest structure of collider stratification bias. In other applications and depending on the conditions of a given experiment, other variables may play a role in the data generation process and should be included in the DAG.

Second, while we show how collider stratification bias can arise in the presence of animal attrition, the magnitude and direction of this bias depend on the parameters included in the structural causal model and the assumed functional relationships between the variables. It was challenging to find realistic parameters for our causal model since animal studies do not typically report all required parameters (e.g. relationship between initial and final infarct volume by treatment group, relationship between initial infarct volume and welfare, or conditional variances needed to define realistic distributions of the error terms).

We assumed the structural causal equation for the final infarct volume was linear with normally distributed errors. Due to this assumption, the conditional causal effect estimated using the adjusted approach also represents an unbiased estimate of the marginal causal effect if no animal had died, which may not generally be the case. Results from randomized experiments without adjustment typically target marginal effects, but arguments have also been raised for the statistical efficiency and relevance of conditional effects.^1^ This is also the reason why the adjusted approach in our application yielded more precise estimates compared with the oracle approach. In the real world, it is plausible that the treatment has an effect on the outcome that varies across animals. In the presence of such effect measure modification (or if the interest lies in a non-collapsible effect measure), more complex statistical methods, such as g-methods,^1^ should be employed to adjust for the selection bias.

We also assumed the relationship between initial and final infarct volumes was linear in the treatment groups. We did not consider more complex scenarios, for example, in which the infarct volume changes over time at different rates based on its initial value. Taken together, parameter choices as well as the assumed linearity of cause-effect relationships in the DAG limit the generalizability of the bias estimates we have reported. The actual magnitude and direction of the collider stratification bias varies for each unique animal disease model, experimental setup and setting.

Third, it is possible that measurement error exists in the real-world. For example, some detection methods (e.g. T2-weighted/diffusion-weighted magnetic resonance imaging) may not be sensitive enough to reliably detect the full initial infarct volume in the early stages after occlusion. When equipment or methods to perform more accurate measurements are not available and the initial infarct volume is measured with error, adjusting for it, for example in a statistical model, will still be preferable (analogous to adjusting for surrogate confounders^1^), although not sufficient to completely mitigate the collider stratification bias introduced by animal attrition.

Fourth, we assumed independent errors in our structural causal model. This assumption implies untestable “cross-world independencies”.^1^ However, we emphasize that the illustrated results and the mechanism of the collider stratification bias hold also without making any cross-world assumption.^1^

Fifth, we acknowledge that to adjust for the selection bias during the analysis phase, it is necessary to correctly model the relationship between the variables. In our didactic example, we knew that the model was correctly specified. However, whether a model is correctly specified is not generally known. Using a model that misspecifies the functional relationships between variables can introduce bias. This problem presents a substantial challenge in preclinical research considering the typically low sample sizes.

Lastly, we opted to exclude the small number of effect estimates in the naive and adjusted approaches that could not be computed due to too few animals in one particular stratum. We acknowledge that excluding these missing estimates may have introduced some bias in the calculated means and quantile ranges.

### Implications

DAGs provide an overview of the complex interplay between variables and serve as a valuable tool to formulate *a priori* hypotheses about the underlying data generating processes. By visually representing these hypotheses, DAGs aid researchers in the detection of potential biases and justify design and analysis choices.^1,22^ As we have shown, DAGs can be employed to 1) detect the potential presence of collider stratification bias before the experiment is run, and 2) identify variables worth measuring since they would be needed to detect and/or mitigate this bias in the analysis phase. Bias mitigation may require the use of statistical models and, therefore, rely on additional assumptions (e.g. linearity and distributional assumptions).^1^ We believe DAGs are particularly well-suited for inclusion into pre-registration of preclinical experiments, especially to justify the measurement of and adjustment for specific variables in cases when biases are suspected.

Our results illustrate how striving for low animal attrition during the course of an experiment can help preserve internal validity in preclinical research. Since higher animal survival probabilities can improve internal validity by reducing selection bias, laboratory and operation-related procedures should be carefully considered when determining whether a particular animal model is suitable.^40,41^ Reputational concerns for the experimenter and the misconception that lost animals do not contribute relevant evidence (and do not need to be reported) both hinder transparency and may explain the slow uptake and insufficient reporting of animal attrition in the preclinical literature.^9,11,42^ Higher attrition rates do not necessarily reflect skill or procedural deficiencies, especially for high-severity disease models, in which euthanasia to minimize animal suffering may be prioritized over maintaining low attrition. However, when experimental conditions make selection bias likely, which can be anticipated prior to performing the study, it raises the question whether the (biased) experimental results, which may lead to further waste down the translation pipeline, have scientific value,^39^ especially if the bias is not addressed.

We advocate for a perceptual shift towards describing the observable differences in relevant characteristics of lost and retained animals. This can reveal valuable insights into potential inferential limitations, particularly, when there is higher attrition in the treatment relative to the control group.^35,43^ The characteristics of lost animals within each experimental group should be systematically documented and published alongside the study results, which aligns with the ARRIVE guideline recommendations.^43^

Multidimensional assessments of animal welfare should include measurements of relevant causes of the animal welfare status (e.g. initial infarct volumes). Explicitly reporting details about these variables and their association with animal welfare and the outcome could strengthen the knowledge about their role in the causal processes in the animal model and be informative to other researchers working with the same model.

Finally, we focus on the potential impact of collider stratification bias for the ischemic stroke *in vivo* model. In a methodological review of articles published between 2006–2016, experimental stroke research was found to be unique in showing improvements in quality and methodological rigor compared with other cardiovascular research areas.^44^ We want to emphasize that our findings likely also extend to other *in vivo* disease models as well as *in vitro* research.

## Conclusions

Even when conducting randomized, controlled, standardized laboratory animal experiments, researchers should be aware of the potential for selection biases that may pose a threat to internal validity. In our study, we have explored the mechanisms behind collider stratification bias, a specific type of selection bias. Though well-described in the field of epidemiology, we have demonstrated why collider stratification bias should be a concern in preclinical studies involving animal models, particularly those with severe side effects and high post-randomization attrition. We focused on scenarios in which the treatment had no actual effect on the outcome, but the researcher would have (falsely) observed one due to this type of selection bias. Collider stratification bias can affect the conclusions drawn from such experiments, in turn influencing the direction of subsequent translational research. Our findings emphasize the importance of considering and addressing selection biases in preclinical research to improve the validity of study results, contributing to more accurate and reliable scientific discoveries.
